# High-Resolution Quantification of Hepatitis C Virus Genome-Wide Mutation Load and Its Correlation with the Outcome of Peginterferon-Alpha2a and Ribavirin Combination Therapy

**DOI:** 10.1371/journal.pone.0100131

**Published:** 2014-06-20

**Authors:** Weihua Wang, Xiaoan Zhang, Yanjuan Xu, George M. Weinstock, Adrian M. Di Bisceglie, Xiaofeng Fan

**Affiliations:** 1 Division of Gastroenterology & Hepatology, Department of Internal Medicine, Saint Louis University School of Medicine, St. Louis, Missouri, United States of America; 2 Saint Louis University Liver Center, Saint Louis University School of Medicine, St. Louis, Missouri, United States of America; 3 The Genome Institute, Washington University School of Medicine, St. Louis, Missouri, United States of America; 4 Wuhan Center for Tuberculosis Control, Wuhan, Hubei, China; Kaohsiung Medical University Hospital, Kaohsiung Medical University, Taiwan

## Abstract

Hepatitis C virus (HCV) is a highly mutable RNA virus and circulates as a heterogeneous population in individual patients. The magnitude of such population heterogeneity has long been proposed to be linked with diverse clinical phenotypes, including antiviral therapy. Yet data accumulated thus far are fairly inconclusive. By the integration of long RT-PCR with 454 sequencing, we have built a pipeline optimized for the quantification of HCV genome-wide mutation load at 1% resolution of mutation frequency, followed by a retrospective study to examine the role of HCV mutation load in peginterferon-alpha2a and ribavirin combination antiviral therapy. Genome-wide HCV mutation load varied widely with a range from 92 to 1639 mutations and presented a Poisson distribution among 56 patients (Kolmogorov-Smirnov statistic  = 0.078, p = 0.25). Patients achieving sustained virological response (n = 26) had significantly lower mutation loads than that in null responders (n = 30) (mean and standard derivation: 524±279 vs. 805±271, *p* = 0.00035). All 36,818 mutations detected in 56 patients displayed a power-law distribution in terms of mutation frequency in viral population. The low-frequency mutation load, but not the high-frequency load, was proportional firmly to the total mutation load. In-depth analyses revealed that intra-patient HCV population structure was shaped by multiple factors, including immune pressure, strain difference and genetic drift. These findings explain previous conflicting reports using low-resolution methods and highlight a dominant role of natural selection in response to therapeutic intervention. By attaining its signatures from complex interaction between host and virus, the high-resolution quantification of HCV mutation load predicts outcomes from interferon-based antiviral therapy and could also be a potential biomarker in other clinical settings.

## Introduction

As a result of error-prone replication and high turnover rate, RNA viruses in persistent infection circulate as a heterogeneous population, sometimes referred as viral quasispecies [1]. The structure of such heterogeneous populations, as shaped theoretically by interactions between host and viruses, may reflect intrinsic pathophysiological status of underlying diseases and may act as a virulence factor to affect disease progression [2]. Given the broad clinical spectrum of resulting diseases as seen with human immunodeficiency virus (HIV) and hepatitis C virus (HCV), heterogeneous viral population structure, measured either by diversity or diversification, has been studied extensively for potential links with disease traits in various clinical settings, including disease progression and responses to antiviral therapy. However, data accumulated thus far appear to be conflicting [3]. With the introduction of next-generation sequencing, it is rapidly appreciated that a large body of viral mutations, due to their low frequency, may be beyond the detection of common methods, including cloning and Sanger sequencing [4]. In the current study, we have first built a technical platform for genome-wide quantification of HCV mutation load at a one percentage resolution of mutation frequency in viral population. Using patient samples from the well-characterized the Hepatitis C Antiviral Long-term Treatment against Cirrhosis (HALT-C) Trial [5], a retrospective study was then conducted to explore the role of viral population structures in HCV antiviral therapy together with evolutionary and clinical interpretations.

## Materials and Methods

### Patient samples

Sixty-five serum samples from patients with chronic HCV 1a infection were used in the current study. Of them four serum samples were from a completed study that investigated the role of intra-patient HCV population diversity through Sanger sequencing of 15 clones derived from partial HCV envelope region, which spanned the HCV hypervariable region 1 (HVR1) (for simplicity, named HVR1 diversity) [6]. These samples were selected based on their HVR1 diversity, representing high, moderate and low points on its exponential distribution curve [6]. Serum sample H77, the prototype of HCV 1a in the USA, was kindly provided by Dr. Robert Purcell (NIH). The HVR1 diversity of H77 was measured using the same method as described previously [6]. These five samples were used to estimate potential bias associated with the long RT-PCR as well as Roche/454 sequencing depth that reaches quantitative saturation of mutation load at the resolution of 1% mutation frequency.

The remaining 60 serum samples were requested from HALT-C trial [5]. Samples were collected from patients prior to 48-week full-dose combination peginterferon/ribavirin antiviral therapy [5]. Among them 30 patients achieved sustained virological response (SVR) and the other had null response as determined in the parent study. To minimize the role of demographic and other factors in final data interpretation, these patients were well matched in terms of sex (all male), ethnic background (all Caucasian), age, body mass index (BMI) liver fibrosis and necroinflammation estimated by Ishak scoring system [7] (Table S1). All patients had HCV genotype 1a infection, as determined by line probe assay (Innogenetics, Belgium) in the parent study. Upon receiving samples, genotype was further confirmed by phylogenetic analysis of a HCV NS5b region through direct amplicon sequencing. HCV RNA titers in all patients were measured using Roche Amplicor HCV Monitor (version 2.0). Of sixty patients, 54 had IL28B genotype data available from the parent study (Table S1).

### Ethics Statement

For collection of serum samples, a written informed consent was obtained from each patient and was approved by local Institutional Review Board in the parental studies [5]. The research protocol for the use of these patient samples was reviewed and approved by the Saint Louis University Institutional Review Board (IRB protocol: SLU15565).

### RNA extraction, long RT-PCR and 454 sequencing

Total RNA from each serum sample was extracted from 280 µL serum and eluted into 60 µL Tris buffer (pH 8.5) using QIAamp Viral RNA Mini kit (Qiagen, Valencia, CA). Long RT-PCR was then applied to amplify a 9022-bp amplicon spanning approximately 96% HCV genome using an optimized protocol as we described previously [8]. In brief, 10.6 µL of extracted RNA was mixed with 9.4 µL RT matrix consisting of 1x SuperScript III buffer, 10 mM DTT, 1 µM QR2 (reverse primer), 2 mM dNTPs, 20 U of RNasein Ribonuclease Inhibitor, 200 U of SuperScript III (Life technologies) and 5 U AMV (Promega). After incubation at 50°C for 75 min, five microliters of RT reaction was applied for the first round of PCR that contained 1.25 mM Mg(OAc)_2_, 1x XL PCR buffer, 2 mM dNTPs, 0.4 µM Trnc-21, 0.4 µM of each primer (WF33 and QR2), and 2 U rTth XL DNA polymerase (Applied Biosystems). Cycle parameters were programmed as 94°C for 1 min followed by the first 10 cycles of 94°C for 30 sec and 65°C for 9 min and final 20 cycles in which the annealing/elongation temperature was reduced to 60°C for 9 min with a 3-sec autoextension at each cycle. Two microliters of the first round of PCR product was used for the second round amplification with primers WF5 and WR55. Cycle parameters were the same as the first round PCR except for the annealing/elongation temperature was changed to 72°C in the first 10 cycles and 68°C in the last 20 cycles. Product at expected size was gel-purified using QIAEX II Gel Extraction Kit (Qiagen). Approximately 3 to 5 µg of purified product was subjected to the construction of fragment library with Rapid Library Preparation Kit (Roche Applied Science), followed by 454 sequencing on GS/FLX Titanium platform.

### Sequence data analysis

Raw sequence data in standard flowgram format (sff) were converted into fasta and qual files using sffinfo command. The sfffile command was used for random data extraction in simulation analysis. Raw sequence reads in fastq format, combined from fasta and qual files, were first filtered in PRINSEQ (v0.19.5) for quality control, including read length ≥70 bp, mean read quality score ≥25, low complexity with DUST score ≤7, ambiguous bases ≤1% and all duplicates [9]. Filtered reads were mapped onto the consensus sequences using a gapped aligner Bowtie 2 [10]. Because HCV genotype 1a forms two separate clusters in phylogenetic trees [8], [11], the consensus sequence for each clade was generated from non-redundant full-length HCV 1a sequences deposited in Los Alamos HCV database (clade 1 = 271; clade 2 = 124) [12]. The clade attribute of each patient sample was determined by phylogenetic analysis based on partial domain or NS5b. Mapped files were then converted into binary format (BAM), sorted and indexed in SAMtools [13], followed by local realignment and base quality recalibration in Genome Analysis Toolkit (GATK) [14]. The latter used reference SNP (single nucleotide polymorphism) files that were produced by combined data from each HCV 1a clade. Base coverage was then computed with BEDTools [15]. Next, by converting post-alignment BAM files into mpileup format in SAMtools, the consensus sequence for each patient sample was called in VarScan (v2.2.3) with the settings of ≥100x base coverage, ≥25 base quality at a position to count a read and ≥50% mutation frequency [16]. Entire pipeline was repeated using individual consensus sequence from each patient sample. Both mutations and indels (insertions and deletions) were called at each position in VarScan with the same settings except for the mutation frequency set at 1%. These indels were confirmed by manual check using the Integrative Genomics Viewer (IGV) [17]. Finally, the nature of each mutation, either synonymous or nonsynonymous, was determined using a custom script [18].

### Distribution pattern analysis

Histograms from either mutation number or frequency were estimated for distribution patterns (power law, lognormal, exponential and Poisson) using Clauset's method [19]. Significance (*p* value) of a given pattern evaluated by maximum likelihood approach was calculated through the goodness-of-fit test based on the Kolmogorov-Smirnov (KS) statistic and likelihood ratios. Consequently, null hypothesis is rejected with the *p* value <0.1 [19]. The test also provides an estimate of the lower cut-off (χ_min_) for the scaling region. All analyses were done with poweRlaw package in R [20].

### Sliding window analysis

The mutation load was measured as the number of mutations or Shannon Entropy that takes into the account of mutation frequency [6]. Over the HCV coding region from Core to NS5b, the mutation load was counted through sliding windows, size = 300 bp, overlap = 100 bp. Statistical significance at each window was determined between SVR and null responders.

### Recovery of structural HCV HVR1 variants

**T**he HVR1 on the 5′ end of HCV E2 is the most variable region under immune selection and/or viral compartmentation. For each patient, all structural variants over a 300-bp window spanning the HVR1 were recovered by the local analysis in program Shorah [21]. Because 300-bp window size is well below the average read length (444 bp) in the current study, the local analysis gives the most reliable estimation that is free of any reconstruction steps in either model-dependent or model-independent manner. Due to highly variable nature of the HVR1, patient-specific consensus sequences were used in Bowtie 2 mapping under the relaxed mapping stringency at f(x) = 10+8ln(x) for functional score setting [10]. Indexed BAM file from the output of Bowtie 2 and patient-specific consensus sequence were then used as the input of Shorah. Each structural HVR1 variant recovered from the local analysis came with a posterior probability and average number of reads supporting the variant. Only structural variants with posterior probability >90% were considered as an authentic recovery [21].

### Phylogenetic analysis

This analysis was used to explore potential association between nucleotide sequences (genotype) and disease traits (phenotypes) through the simulation of phylogenetic topologies [22]. Due to genome-wide speciation among HCV genotypes, subtypes and clades, the analysis was performed within each HCV 1a clade under two phenotypes, i.e., response patterns to antiviral therapy (SVR vs. null responders) and mutation load (high vs. low). Under these settings, nearly full-length HCV 1a coding region was scanned through a sliding window (size = 300 bp, overlap = 99 bp). The analytical pipeline was similar to what we described previously [23]. In brief, the best-fit nucleotide substitution model for all HCV domains was determined through JModelTest [24]. Bayesian Markov chain Monte Carlo (MCMC) phylogenetic trees were simulated in BEAST package under selective model (GTR+G) as well as additional parameter settings, including a relaxed molecular clock, a Bayesian skyline coalescent prior and a total run of 10 million generations to reach relevant parameter convergence as estimated by Tracer [25]. The inferred MCMC trees, after discarding the first 10% trees as burn-in, were used as the input to estimate the strength of HCV 1a strain clustering in terms of response pattern or mutation load with 1000 replications in BaTS [22]. Based on empirical and simulated data, *p* values of both the association index (AI) and the parsimony score (PS) less than 0.01 were considered as having statistical significance [22], [24]. Other phylogenetic tree constructions were done with MEGA program (version 5.2) [26].

### Statistical analysis

Except for those statistical analyses stated specially, other comparisons between SVR and null responders were done with either two-tailed, unpaired Student's t test or Fisher's exact test. Multivariate Analysis of Variance (MANOVA) among host and viral factors possibly affecting the treatment outcome or the HCV mutation load was executed with IBM SPSS software (version 20). Data were expressed as mean value and standard derivation (SD) and *p*<0.05 was considered statistically significant.

### Data availability

Raw sequence data in fastq format from all 61 patient samples were archived in NCBI Sequence Read Archive (SRA) under SRA accession number SRP035878.

## Results

### Genome-wide quantification of HCV mutation load

**Figure 1:**
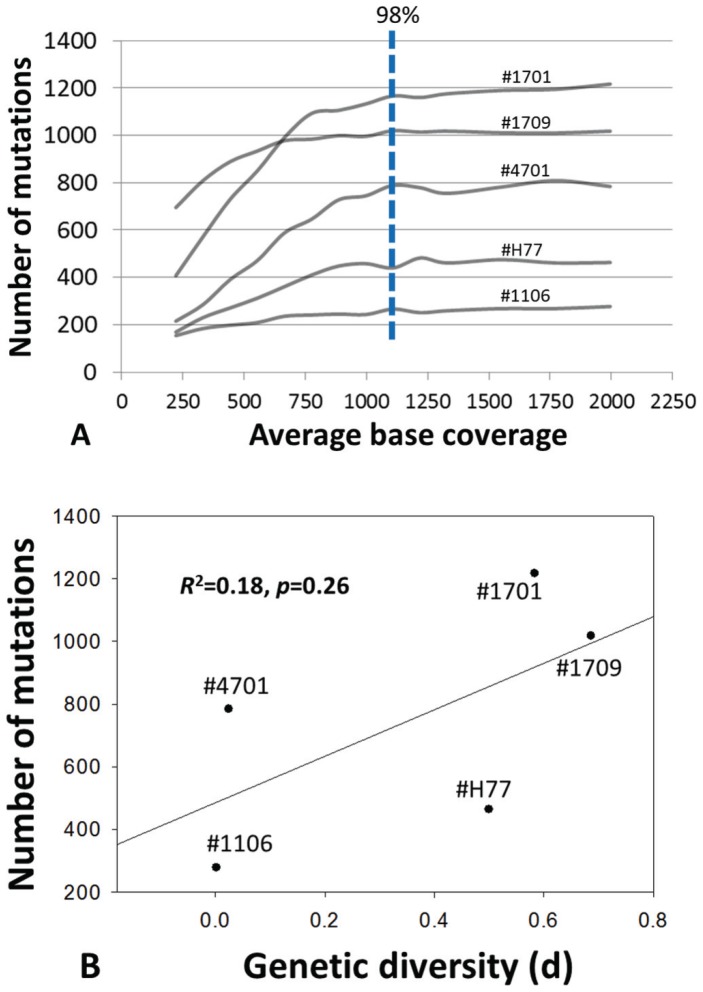
Simulation of the sequencing depth required for stable quantitation of HCV mutation load at a resolution of 1% mutation frequency in population. Five patient samples with known intra-population genetic diversity were subject to 454 sequencing. Starting at an average base coverage of 1100x, the genome-wide HCV mutation load became stable with overall 98% saturation (A) and had a weak correlation with genetic diversity measured by cloning and sanger sequencing (*R* ^2^ = 0.18, *p* = 0.26) (B).

The quantification of HCV mutation load was achieved by the integration of the long RT-PCR technique [8], 454 sequencing and bioinformatic pipelines. All viral mutations with the minimum of 1% frequency in population were counted over the 9022-bp amplicon, spanning nearly 96% of the HCV genome from HCV 5′UTR to NS5B region. To determine an appropriate sequencing depth for comparable quantification of mutation load among patients, we applied the method for five HCV patient samples with known intra-population HCV HVR1 diversity [6]. The long RT-PCR amplicons from these patients had an output of 567,904 reads with an average read length at 393.3 bp, which was translated into an average sequencing depth at 4946x. Through multiple random data extraction, we quantified mutation load at various sequencing depths. For each sample, the mutation load became stable with an average of 98% saturation upon approaching the depth of 1100x (Figure 1A). As five samples were selected in terms of their representative genetic diversity on an exponential curve (low, moderate and high) [6], proportional association between the genome-wide mutation load and the genetic diversity was weak (*R* ^2^ = 0.18, *p* = 0.26) (Figure 1B). A theoretical calculation of per base sequencing depth ≥1100x was applied in subsequent retrospective studies.

**Figure 2:**
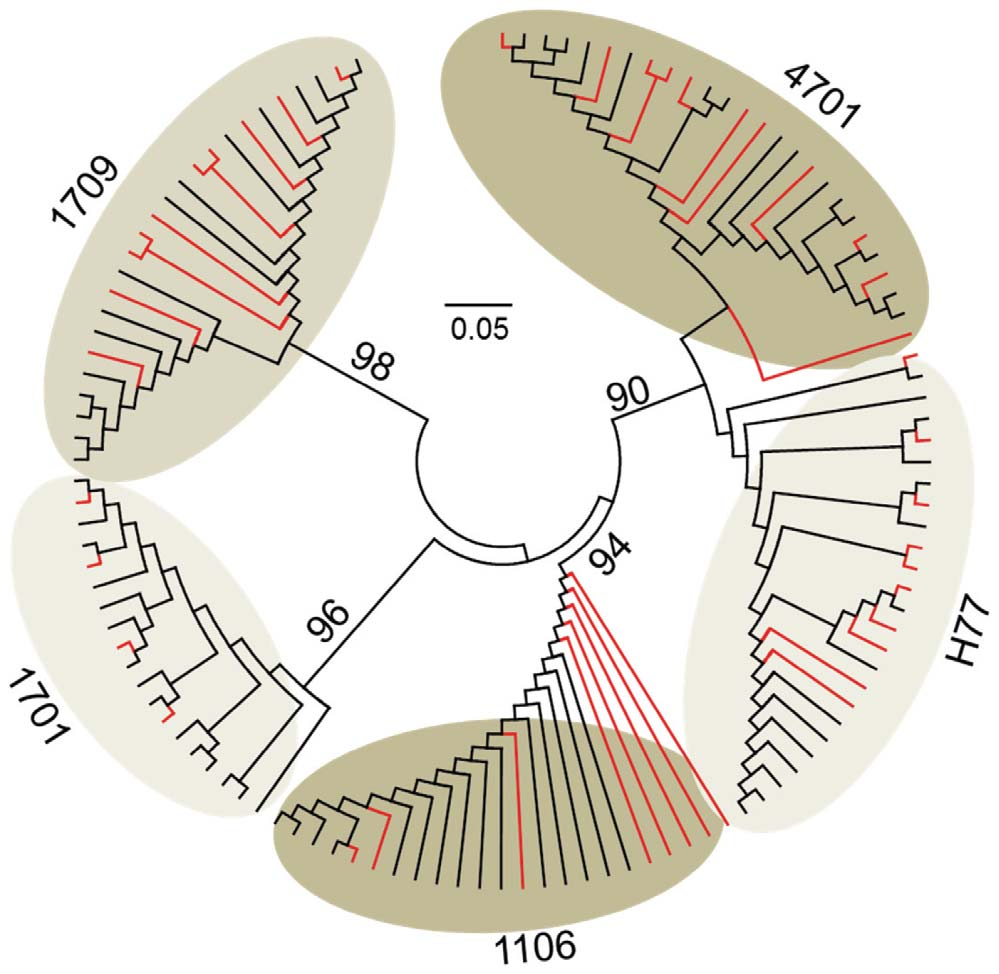
Neighbor-joining tree of HCV HVR1 variants recovered by either 454 sequencing (red) or cloning/Sanger sequencing (black) in five patient samples. The tree was constructed under maximum composition likelihood model with bootstrap support (percentage) shown on major branches.

**Figure 3:**
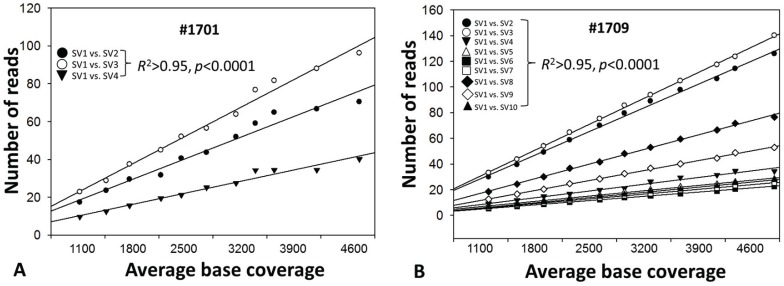
Linear regression analysis of the correlation among reads supporting each HVR1 structural variant (SV) recovered at various sequencing depths in samples #1701 (A) and #1709 (B). Data were extracted randomly in a range of sequencing depths starting at 1100x, a saturation point for the mutation load. At the depth of 1100x, samples #1701 and #1709 had 4 and 10 HVR1 structural variants, respectively, which were not changed up to the depth of 4600x. In each sample, the correlation between HVR1 structural variant 1 (SV1) and the other variants (SV2 through SV3 in #1701 and SV2 through SV10 in #1709) was highly stable throughout the sequencing depths (all *R* ^2^>0.95, *p*<0.0001).

All structural variants over a 300-bp HCV domain, spanning the HCV HVR1, were recovered from each of five patients at theoretical sequencing depth of 1100x. The HVR1 variants detected from either 454 sequencing or cloning/Sanger sequencing showed high phylogenetic consistency at the lineage level (Figure 2). No additional HVR1 structural variants were found at the depth of 2500x in all samples and even up to 4600x for samples #1701 and #1709, which had the deepest base coverage. Notably, the simulation experiment with these two samples showed highly stable ratios among the read numbers supporting each HVR1 structural variant over a range of depths (Figure 3).

### Low mutation load is associated with sustained virological response (SVR)

**Figure 4:**
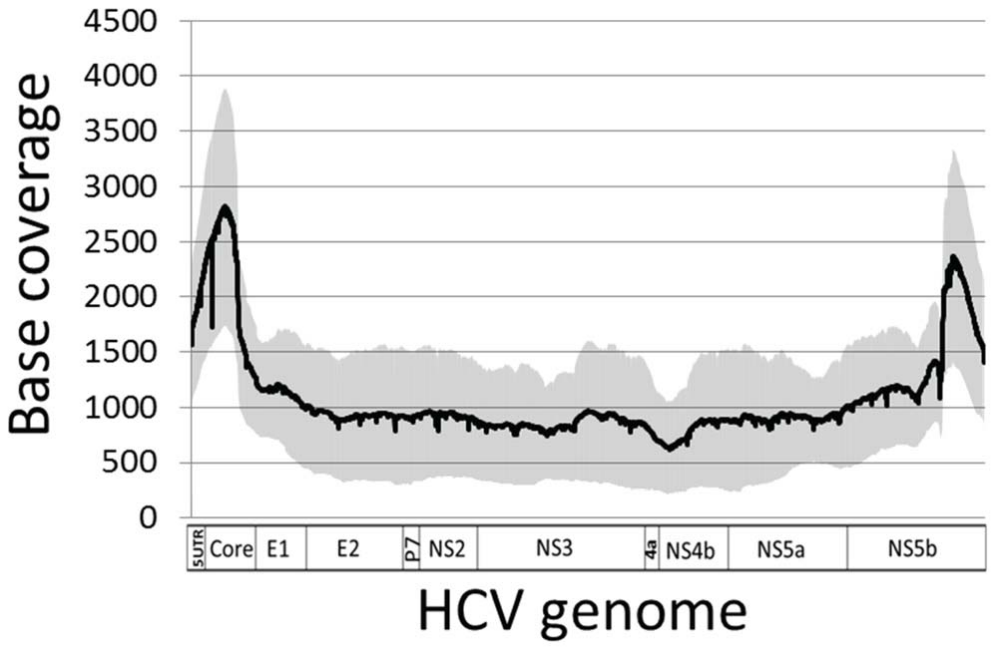
Average base coverage over 9022-bp amplicon after read quality control. The amplicon covers 96% HCV genome from 5′UTR to NS5b as indicated at the bottom. Grey area represented standard derivation of the base coverage at each HCV genomic position.

**Figure 5:**
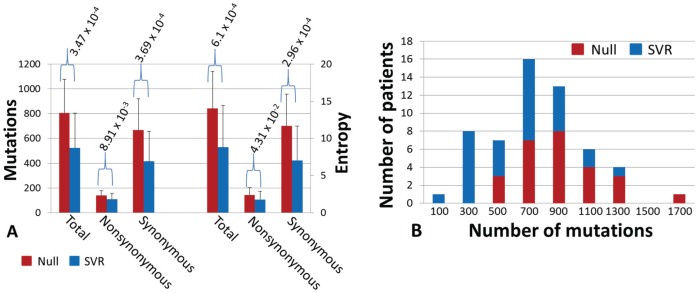
Comparative analysis of the mutation load, either average number of mutations per patient (left side) or normalized entropy (right side), between SVR and null response groups (A). The analysis was done with either synonymous, nonsynonymous or the combination (A). The histogram of total mutation number, fitting a Poission distribution, gave a more intuitive observation with respect to its relevance to the outcomes of HCV antiviral therapy (B).

The method established was used to examine potential association between mutation load and the outcome of HCV antiviral therapy. A total of 60 patients who underwent 48-week peginterferon α-2a and ribavirin combination therapy were selected from the HALT-C trial. After the exclusion of samples that failed long RT-PCR amplification (n = 3) or the quality control of 454 library preparation (n = 1), 454 sequencing of the remaining 56 patient samples generated 1,454,077 reads with an average read length at 444 bp, thereby an average theoretical sequencing depth calculated at 1257x. After read quality control, average 95.4% and 99.7% mapping rates were achieved with the consensus sequences from HCV 1a clades and individual strains, respectively. Actual per base coverage after read quality control was 1094±577x (Figure 4). There was no difference between SVR (n = 26) and null responders (n = 30) with regard to the sequencing depth and the read length (data not shown). For all 56 patients, we identified 36,818 mutations and 1,616 indels at the resolution of 1% frequency in population. One patient had a large in-frame 1455-bp deletion in HCV structural region spanning E1 through p7 (Figure S1), which was subsequently confirmed by the lack of RT-PCR amplification using primers located in this domain (data not shown). The mutation load, either the number of mutations or the normalized entropy, varied widely among patients, ranging from 92 to 1639 and 1.19 to 24.9, respectively. Notably, the mutation load was significantly lower in the SVR compared to the null responders, 524±279 vs. 805±271, *p* = 3.47×10^−4^ (mutation) and 8.8±5.6 vs. 14±5, *p* = 6.1×10^−4^ (entropy) (Figure 5A). Among 35 patients with unfavorable IL28B genotypes (CT and TT), average mutation load per patient remained different significantly between SVR and null responders (762.3±228.6 vs. 618.3±171.4, *p* = 0.01).

In contrast with an exponential distribution of HCV genetic diversity measured by cloning and Sanger sequencing [6], the histogram of mutation load presented a Poisson distribution (KS = 0.078, *p* = 0.25), which provided an intuitive view for the correlation between HCV mutation load and treatment responses (Figure 5B). Finally, no overall difference was observed with regard to total numbers of the indels between SVR (27.4±9.6) and null groups (30.1±14.8) (*p* = 0.44). However, sliding window analysis showed that three domains, one at NS4a and the others at the 5′ end of NS5B, had significantly higher numbers of indels in SVR than that in null responders (Figure S2).

### Viral population structures stratified by mutation frequency and mutation nature

**Figure 6:**
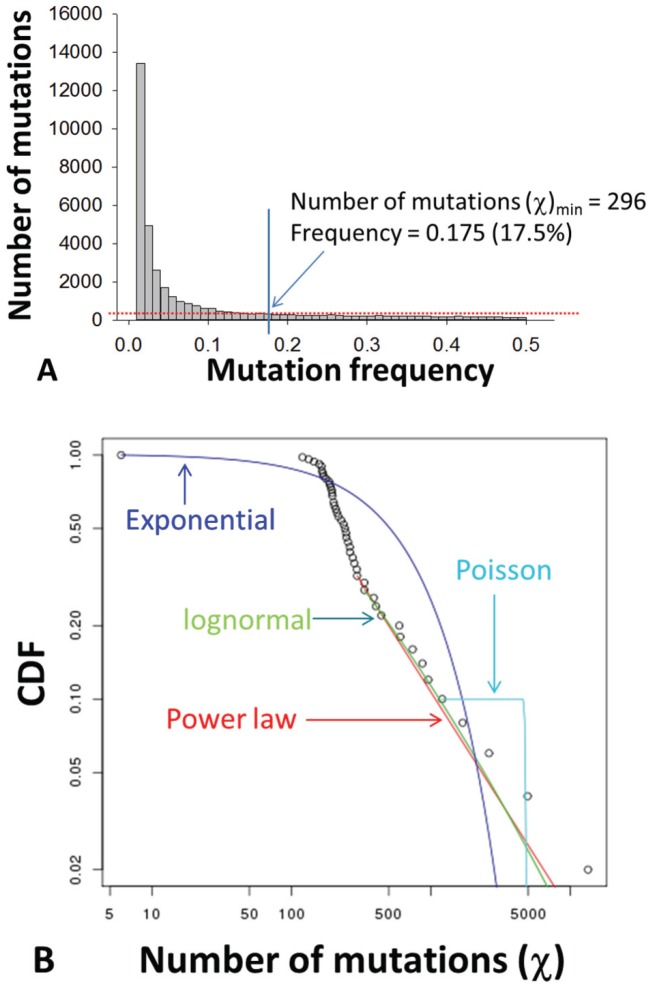
The mutation frequency-based histogram of a total of 36,665 mutations identified in 56 patients (A), which fitted in with a power law distribution (B) with the low-bound point (χ_min_) at 296, corresponding with a mutation frequency at 17.5% (A). CDF, cumulative distribution function.

**Figure 7:**
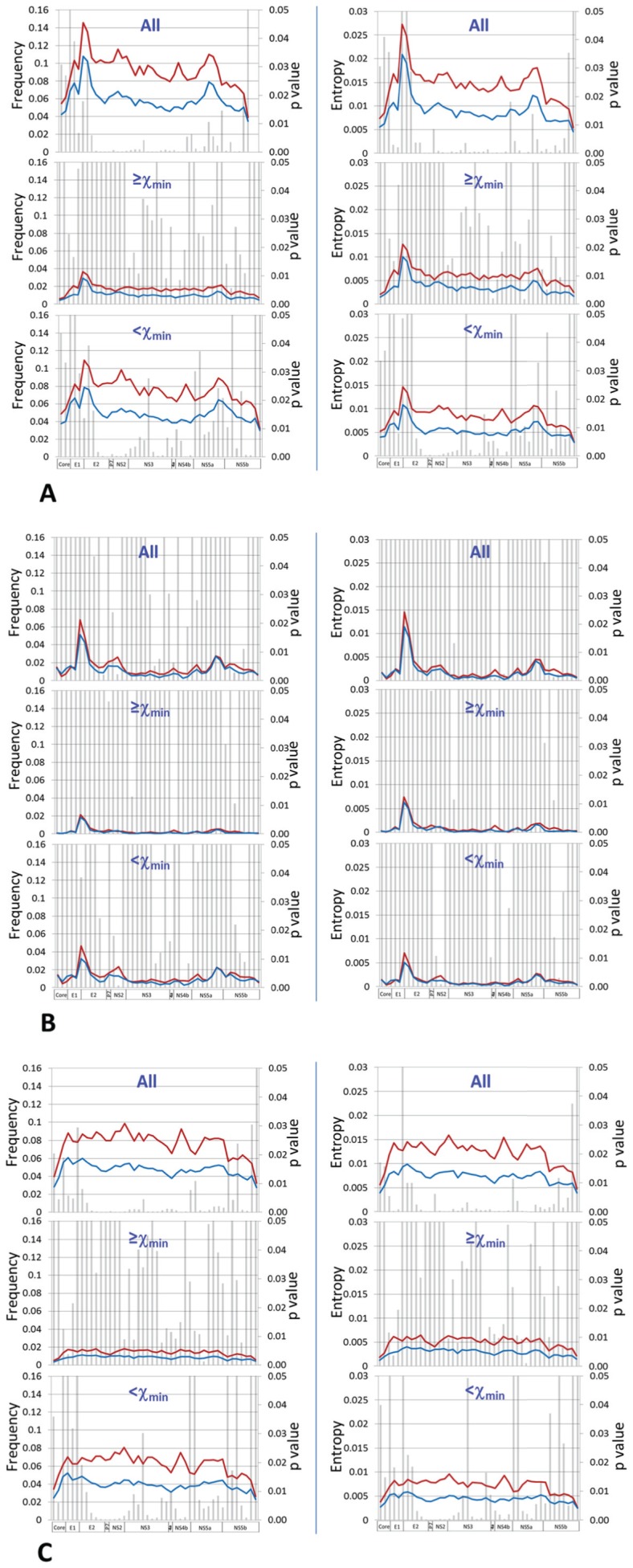
Comparative HCV genome-wide sliding window analyses (window size = 300 bp; overlap  = 99 bp) between SVR (blue) and null response group (red) according to mutation nature, including total mutations (A), synonymous mutations (B) and nonsynonymous mutations (C). Mutations within each category were analyzed separately based on χ_min_ translated value (17.5%), i.e., low or high mutation frequencies. For each window, average mutation frequency per site (left column) and normalized entropy (right column) were compared for statistical significance as represented by bars.

Of 36,818 mutations detected, most had low frequencies. The histogram fitted either a lognormal (KS = 0.08, *p* = 0.78) or a power law distribution (KS = 0.09, *p* = 0.84) (Figure 6). A slightly higher *p* value, as defined in Clauset's approach [19], suggested the better acceptance of power law distribution for our data. The low-bound point (χ_min_) was calculated at 296, corresponding with a mutation frequency at 17.5% (Figure 6A). Stratified by either mutation frequency based on χ_min_ (high vs. low) or mutation nature (synonymous vs. nonsynonymous), the patterns of mutation distribution gave a deep insight into viral population structure in a genome-wide manner. Over the HCV coding region from Core to NS5b that accounted for 36,665 mutations (99.6% of total mutations), both mutation frequency and entropy had essentially the same patterns in all stratifications (Figure 7). The magnitude of differences was not apparent between SVR and null responders in nonsynonymous but not in synonymous mutations (Figure 7). The former had apparent mutation peaks, one in the E2 and the other in the NS5a (Figure 7B), which were all lost in the patterns of synonymous mutations (Figure 7C). Finally, statistical significance was compromised in most viral domains in the patterns with either nonsynonymous or high-frequency (≥17.5%) mutations (Figure 7).

### Contribution of viral and host factors to the mutation load

By a Poisson distribution pattern of the mutation load among 56 patients, the high or low mutation load was assigned using the mean value (n = 654) of the total mutation number. Owing to a well-matched selection of patients (Table S1), two groups with either low or high mutation load had no difference on demographic factors (race, sex, age and BMI) and disease status (Ishak scores). Comparative analysis was thus focused on viral and host genetic factors, including viral compartmentalization, viral population size, viral strain difference and IL28B genotype. A total of 319 HVR1 structural variants were recovered from 56 patients (Figure S3). The average number of structural HVR1 variants per patient was not different between two groups, 4.96±2.24 vs. 5.07±2.25, *p* = 0.86 (Table 1). Similarly, the mutation load was not apparently associated with HCV RNA titers (6.14±0.46 vs. 6.36±0.38, *p* = 0.051) (Table 1). Of fifty patients with the availability of both mutation load and IL28B genotype data, patients with IL28B CC type had significant low mutation load in comparison with non-CC types (CT and TT), 480±341 vs. 721±221, *p* = 0.004, corresponding with the count of double number of patients with IL28B CC type in the group of low mutation load in spite of the lack of statistical significance (Table 1). Finally, except for the body weight and BMI, MANOVA analysis did not find any significant covariance among host and viral factors in terms of the treatment outcome or the mutation load (all p values>0.2).

**Table 1:** Comparisons of viral and host factors in the context of therapeutic outcomes (SVR vs. null) or mutation load (low vs. high).

Factors			Treatment outcome		Mutation load	
			SVR vs. Null	p value*	Low vs. High	p value*
**Host**	Race		All Caucasian		All Caucasian	
	Sex		All male		All male	
	Age		48.4±4.71 vs. 48±4.3	0.745	48.5±4.27 vs. 47.2±3.97	0.262
	Weight (kg)		86.4±13.1 vs. 90.3±14.1	0.267	91.6±15.24 vs. 86.3±12.2	0.152
	BMI		27.9±3.59 vs. 28.9±3.96	0.291	29.1±4.47 vs. 27.9±3.21	0.256
	Ishak score		3.1±0.6 vs. 3±0.7	0.6	3.04±0.64 vs. 3.14±0.71	0.553
	IL28B CC type		15/27 vs. 1/27	5×10^−5#^	10/25 vs. 5/25	0.217#
**HCV**	HCV genotype		All genotype 1a		All genotype 1a	
	HCV RNA load (log_10_)		6.3±0.41 vs. 6.24±0.49	0.423	6.14±0.46 vs. 6.36±0.38	0.051**
	HVR1 variants		4.96±2.23 vs. 5.07±2.25	0.86	5.04±2.4 vs. 4.96±1.88	0.89
	Genome scan	Full-length	Negative		Negative	
		Domains	Negative		NS5a and NS5b	

*two-tailed Student's t test;

# Fisher's exact test. BMI, body mass index;

**p = 0.063 after controlling other factors in MANOVA (F statistic = 3.62); Scanning indicates an approach to investigate the relatedness between genotypes and phenotypes through calculating statistical significance of phylogenetic clustering.

**Figure 8:**
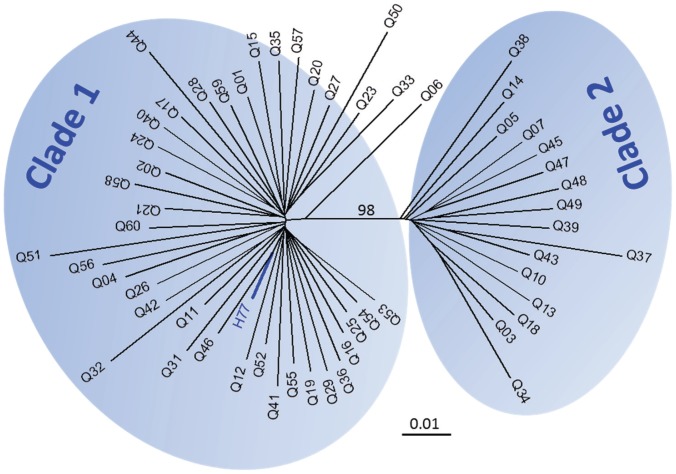
Neighbor-Joining tree of 56 nearly full-length HCV consensus sequences (each 8817 bp in length spanning from HCV Core to NS5b) under maximum composition likelihood model and gamma distribution of rate variation among sites (γ = 0.5). HCV genotype 1a prototype H77 strain was also included (blue branch). All strains were clustered apparently into two clades with 98% bootstrap support.

Next, we determined if any particular HCV 1a strain or viral domains were associated with the mutation load through a phylogenetic approach. On the full-length HCV phylogenetic tree, the strains derived from 56 patients were clustered into two clades, clade 1 (n = 40) and clade 2 (n = 16) (Figure 8). The analysis was thus performed for the clades 1 and 2, respectively. At the full-length HCV genome level, no association between phylogenetic clustering and mutation loads was found in either clade 1 or clade 2 (AI and PS, *p*>0.01, Table S2). However, in a genome-wide domain scan, two domains, domains 34 and 39 respectively located in NS5a and NS5b, showed significant mutation load-dependent clustering in both HCV clades (AI and PS, *p*<0.01, Table S2). Similar analysis showed no relationship in the context of the treatment outcome (Table S2).

## Discussion

**Figure 9:**
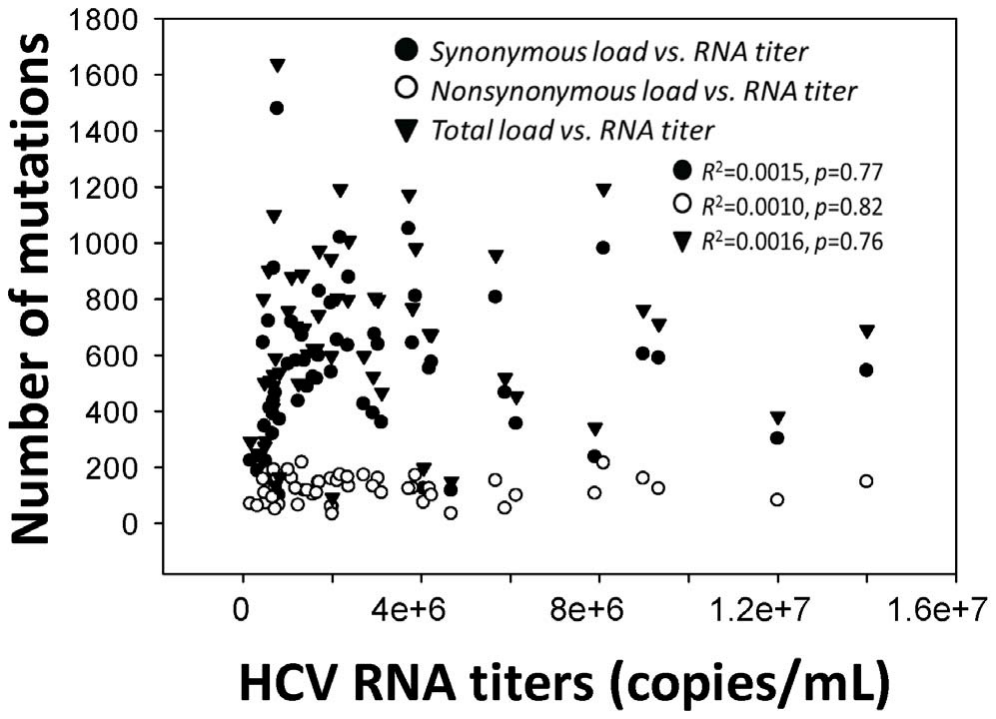
Lack of correlation between individual HCV RNA titers and mutation loads, including synonymous (*R* ^2^ = 0.0015, *p* = 0.77), nonsynonymous (*R* ^2^ = 0.001, *p* = 0.82) and the combination (*R* ^2^ = 0.0016, *p* = 0.76).

In this study, genome-wide quantification of HCV mutation load at a high resolution was achieved through the integration of long RT-PCR, 454 sequencing and bioinformatic tools. RT-PCR of heterogeneous templates is known to be associated with introduction of artificial mutations as well as amplification bias. Recently, several studies reported the use of sequence identifiers in primers for RT or PCR, which allow subsequent monitoring of amplification bias in 454 sequencing data [27], [28]. However, these approaches require an amplicon below the length of sequencing reads and are thus not feasible for long RT-PCR. Under the current experimental settings, we have estimated potential biases that may vary largely among different protocols [29]. First, direct 454 sequencing of DNA templates has a mean error rate at 1.07%, which requires ≥5x read coverage to correct an error [30]. The mean sequencing depth in the current study is 1094±577x after data quality control, equivalent to a read coverage at 5.17x to 16.7x per mutation under 1% resolution of mutation frequency. Thus most errors associated with 454 sequencing should be corrected. Second, the long RT-PCR uses primers located in the conserved regions of HCV genome to allow the full recovery of viral variants [31]. The inclusion of Deep Vent DNA polymerase in the enzyme blend (rTth, XL DNA polymerase) not only facilitates the amplification of nearly full-length HCV genome but also reduces artificial mutations owing to its strong 3′→ 5′ proofreading exonuclease activity. In our previous study, erroneous substitution rate is approximately at 0.13% after 60-cycle PCR cycles without the RT, translating into about 11.7 sites over 9022-bp amplicon [32]. Thus potential PCR-associated errors should have a minimal role on HCV mutation load measured in a genome-wide manner. Such a minimal role is further debilitated through a comparative analytical strategy in the present study. Third, individual patient HCV mutation loads didn't correlate with viral titers (Figure 9), suggesting a minimal effect of the template amount on the mutation load. Finally, in the simulation experiment, the number of HVR1 structural variants and more importantly, the relative ratio of sequencing reads supporting each HVR1 structural variant, were fairly stable over various sequencing depths (Figure 3), indicating the lack of amplification bias at least at the level of structural variants. Indeed, in spite of the circulation as a heterogeneous population, the number of HCV HVR1 structural variants was limited, ranging 1 to 9 variants, and further reduced in terms of phylogenetic lineages (Figure S3). Taken together, while we are unable to map potential bias relevant to individual variants, above observation show that such a bias is unlike to have a role in the genome-wide mutation load that doesn't have a focus on particular sites and mutations.

Applying this method to well-characterized human subjects, we found the first unambiguous evidence that low mutation load in a genome-wide manner is associated with the better response to antiviral therapy. This conclusion remains effective when including only patients with unfavorable IL28B genotypes (CT and TT), one of the strongest single factors to predict treatment outcomes [33]. Thus, the HCV genome-wide mutation load at high resolution appears to be an independent factor to predict the therapeutic results. While excess mutations are detrimental to viral population fitness [34], [35], our data indicates a dominant role of natural selection even in patients with high mutation loads, which maximize the potential of HCV mutational pathways to counteract antiviral drugs, as observed in recent *in vitro* experiment [36].

**Figure 10:**
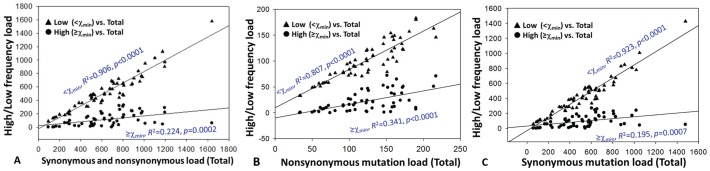
Linear regression analysis of the relatedness between high/low frequency mutation loads and respective total mutation loads, i.e., synonymous and nonsynonymous mutations (A), nonsynonymous mutations (B) and synonymous mutations (C).

In-depth analysis of the mutation load explains previous conflicting data with regard to the predicting value of viral population structure in HCV antiviral therapy. The power-law pattern of mutation histogram had a low-bound point crossed with a mutation frequency at 17.5% (Figure 6), which is beyond the detection limit of common methods like gel shift analysis or cloning and Sanger sequencing [37], [38]. However, it should be noted that conventional cloning and Sanger sequencing is comparable with 454 sequencing for the recovery of structural HCV HVR1 variants at the level of phylogenetic lineages (Figure 2). The low-frequency mutation load, but not the high-frequency, is proportional strongly to total mutation loads of synonymous (*R* ^2^ = 0.923, *p*<0.0001), nonsynonymous (*R* ^2^ = 0.807, *p*<0.0001) or the combination (*R* ^2^ = 0.906, *p*<0.0001) (Figure 10). Consequently, the genome-wide sliding window analysis revealed that statistical significance in most regions were compromised upon exclusive inclusion of high-frequency mutations (>17.5%) (Figure 9). Interestingly, the HCV HVR1, a domain used frequently in viral diversity studies, is less powerful in distinguishing SVR from null responders (Figure 7). Thus conflicting data obtained with common methods appears to be the result of low-resolution of mutation detection.

Our analysis further reveals that multiple forces together shape the viral population structure. Although not absolute, synonymous and nonsynonymous mutations are commonly interpreted as the reflection of selective and neutral forces, respectively [39]. Confirming to this assertion, sliding window analysis of nonsynonymous, but not synonymous mutations, shows an apparent peak in the HVR1, a well-documented region under high immune selection [40]. The higher number of nonsynonymous mutations thus suggests a stronger immune pressure in the SVR group compared to the null responders (Figure 5). Second, among 36,665 mutations detected in HCV coding region from 56 patients, 24,375 (66.48%) are low-frequency synonymous mutations from which statistical significance stems mostly (Figure 7). Given comparable numbers of structural HCV HVR1 variants between SVR and null responders (Table 1), viral compartmentation, often associated with distinct HCV HVR1 variants [41], might play a negligible role in the contribution to observational difference of the mutation load. Therefore genetic drift and its magnitude might be a reasonable explanation for both mutation accumulation and differential mutation loads in SVR and null responders. Third, two HCV domains, respectively located in NS5a and NS5b, showed significant mutation load-dependent clustering (Table S2). The NS5b encodes RNA-dependent RNA polymerase that drives an error-prone viral replication. Mutations in NS5b or nearby regions, together with increasing number of indels (Figure S2), may have a direct effect on strains' intrinsic mutation rates. Lastly, it was interesting to note that the low mutation load was associated with IL28B CC type, one of single strongest predictors in interferon-based HCV antiviral therapy. Taken together, these data may delineate a scenario regarding the generation and modulation of HCV mutation load. Besides direct contribution of nonsynonymous mutations, the HCV-target immunity, both innate and adaptive, modulate viral replication dynamics that affect the strength of genetic drift by coupling with viral intrinsic mutation rates. While molecular mechanisms underpinning these observations remain largely unknown, it is clear that no single factors from either virus or host side could dominate the mutation load in chronic HCV infection. A power law distribution among patients indeed signifies the operation of potential multiple-level hierarchies on the modulation of HCV mutation load.

In conclusion, by establishing a method for genome-wide quantitation of HCV mutation load, the current study explains previous conflicting observation and intensifies a dominant role of natural selection in HCV population in response to interferon-based antiviral therapy. We showed that intra-patient HCV population structure attains the signatures from complex interaction between host and virus. Thereby the high-resolution quantitation of HCV mutation load could be a useful approach to evaluate outcomes in interferon-based antiviral therapy and potentially in other clinical settings.

## Supporting Information

Figure S1 **A graphic illustration of read alignment in patient Q52 against the consensus sequence of HCV genotype 1a clade 1, which showed a 1455-bp large structural deletion from position 1203 to 2657 according to HCV genotype 1a prototype H77 (Genbank accession number AF009606).**
(TIF)

Figure S2 **Sliding window analysis of indels.** Of 1,616 indels identified in 56 patient samples, the sliding window analysis (window size = 300 bp, overlap = 100 bp) showed similar distribution patterns over HCV genome and in most domains, i.e., 42 of 45 windows, statistical significance, represented by bars, was not approached by two-way student's t test between SVR (blue) and null responders (red). Three domains located in NS4a and NS5b had significantly higher numbers of indels in SVR than that in null responders.
(TIF)

Figure S3 **Neighbor-Joining tree of 319 structural variants (each 300 bp) spanning HCV HVR1 domain, which were recovered from read libraries of 56 patients except for patient Q52 who had a large in-frame structural deletion.** Bootstrap test was done with 500 replicates and the support (percentage) was shown at major branches.
(TIF)

Table S1 **An overview of demographic, virological, disease status and genetic information of 60 patients enrolled in the study.**
(DOCX)

Table S2 **Results of BaTS analysis based on either nearly full-length HCV 1a genome (8817 bp) or genome scan.**
(DOCX)
