# Supplementary figures and images for: High-Resolution Quantification of Hepatitis C Virus Genome-Wide Mutation Load and Its Correlation with the Outcome of Peginterferon-Alpha2a and Ribavirin Combination Therapy
Source: PLoS One. 2014 Jun 20;9(6):e100131. doi: 10.1371/journal.pone.0100131 (PMC4065037; doi:10.1371/journal.pone.0100131)

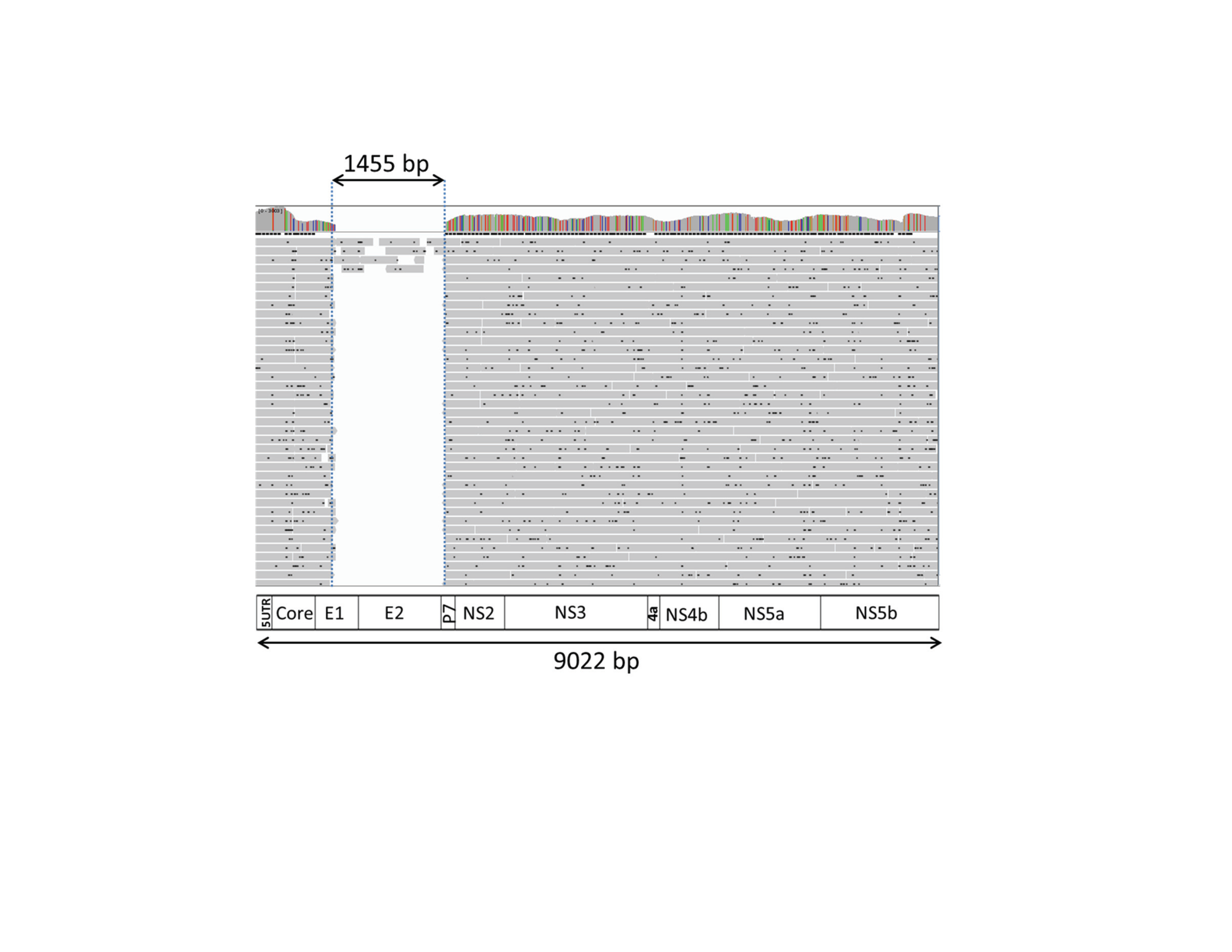

Supplement: Figure S1 — A graphic illustration of read alignment in patient Q52 against the consensus sequence of HCV genotype 1a clade 1, which showed a 1455-bp large structural deletion from position 1203 to 2657 according to HCV genotype 1a prototype H77 (Genbank accession number AF009606). (TIF) [file pone.0100131.s001.tif]

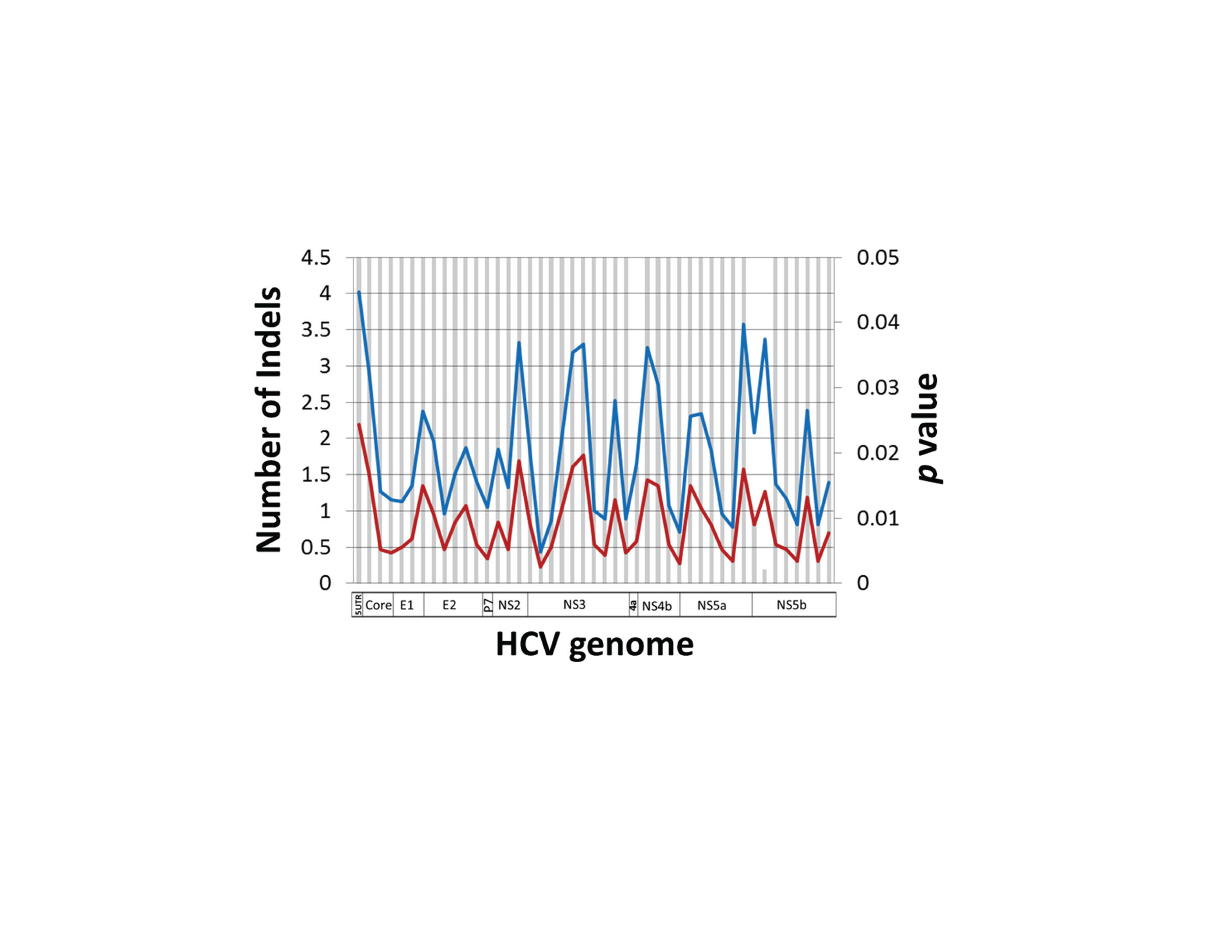

Supplement: Figure S2 — Sliding window analysis of indels. Of 1,616 indels identified in 56 patient samples, the sliding window analysis (window size = 300 bp, overlap = 100 bp) showed similar distribution patterns over HCV genome and in most domains, i.e., 42 of 45 windows, statistical significance, represented by bars, was not approached by two-way student's t test between SVR (blue) and null responders (red). Three domains located in NS4a and NS5b had significantly higher numbers of indels in SVR than that in null responders. (TIF) [file pone.0100131.s002.tif]

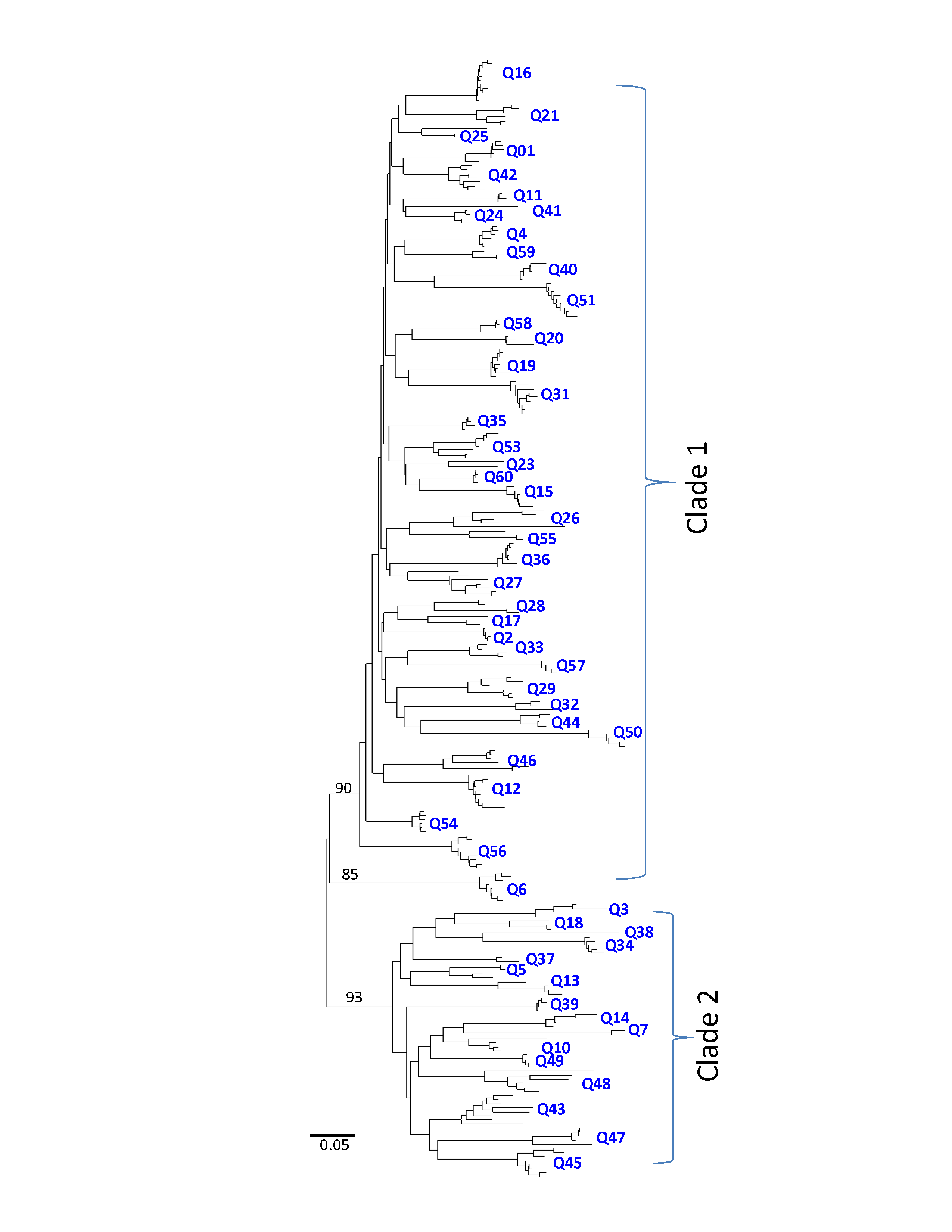

Supplement: Figure S3 — Neighbor-Joining tree of 319 structural variants (each 300 bp) spanning HCV HVR1 domain, which were recovered from read libraries of 56 patients except for patient Q52 who had a large in-frame structural deletion. Bootstrap test was done with 500 replicates and the support (percentage) was shown at major branches. (TIF) [file pone.0100131.s003.tif]
