# Supplementary material for: High-Resolution Quantification of Hepatitis C Virus Genome-Wide Mutation Load and Its Correlation with the Outcome of Peginterferon-Alpha2a and Ribavirin Combination Therapy
Source: PLoS One. 2014 Jun 20;9(6):e100131. doi: 10.1371/journal.pone.0100131 (PMC4065037; doi:10.1371/journal.pone.0100131)
Supplement: Table S1 — An overview of demographic, virological, disease status and genetic information of 60 patients enrolled in the study. (DOCX) [file pone.0100131.s004.docx]

| **Patients** | **Response** | **Age** | **Sex** | **Race** | **Weight** | **BMI** | **HCV genotype** | **HCV RNA** | **IS** | **IL28B** |
| --- | --- | --- | --- | --- | --- | --- | --- | --- | --- | --- |
| 1 | Null | 50 | M | White | 75.4 | 23.53 | 1a | 6.375 | 4 | CT |
| 2 | Null | 45 | M | White | 58.1 | 21.6 | 1a | 6.297 | 3 | TT |
| 3 | Null | 45 | M | White | 100.8 | 31.46 | 1a | 6.621 | 3 | TT |
| 4 | Null | 48 | M | White | 76.7 | 23.94 | 1a | 5.827 | 4 | TT |
| 5 | Null | 46 | M | White | 72.6 | 26.99 | 1a | 6.588 | 3 | TT |
| 6 | Null | 48 | M | White | 75.4 | 25.79 | 1a | 6.228 | 4 | CT |
| 7 | Null | 51 | M | White | 99.6 | 27.88 | 1a | 5.860 | 2 | CT |
| 8 | Null | 52 | M | White | 80.4 | 26.56 | 1a | 7.481 | 3 | CT |
| 9 | Null | 63 | M | White | 78.9 | 27.63 | 1a | 6.276 | 3 | CC |
| 10 | Null | 51 | M | White | 79.2 | 25.86 | 1a | 6.580 | 3 | CT |
| 11 | Null | 51 | M | White | 72.6 | 26.35 | 1a | 6.571 | 3 | CT |
| 12 | Null | 48 | M | White | 97.6 | 27.91 | 1a | 6.137 | 3 | TT |
| 13 | Null | 45 | M | White | 114.5 | 33.82 | 1a | 5.754 | 4 | CT |
| 14 | Null | 58 | M | White | 98.5 | 31.8 | 1a | 5.913 | 3 | CT |
| 15 | Null | 51 | M | White | 106.1 | 37.15 | 1a | 6.093 | 2 | TT |
| 15 | Null | 55 | M | White | 76.1 | 24.57 | 1a | 5.885 | 3 | NA |
| 17 | Null | 42 | M | White | 80.4 | 28.15 | 1a | 6.970 | 2 | NA |
| 18 | Null | 42 | M | White | 71.3 | 24.96 | 1a | 5.838 | 4 | CT |
| 19 | Null | 45 | M | White | 96.2 | 30.71 | 1a | 5.775 | 3 | CT |
| 20 | Null | 46 | M | White | 96.7 | 33.86 | 1a | 6.294 | 4 | TT |
| 21 | Null | 46 | M | White | 96.2 | 31.77 | 1a | 6.470 | 3 | CT |
| 22 | Null | 49 | M | White | 80.8 | 27.63 | 1a | 6.679 | 3 | TT |
| 23 | Null | 43 | M | White | 89.1 | 27.2 | 1a | 6.324 | 3 | CT |
| 24 | Null | 47 | M | White | 86.9 | 24.85 | 1a | 6.433 | 4 | CT |
| 25 | Null | 42 | M | White | 90.5 | 31.31 | 1a | 6.953 | 3 | CT |
| 26 | Null | 49 | M | White | 90.2 | 26.93 | 1a | 6.037 | 3 | CT |
| 27 | Null | 45 | M | White | 69.9 | 22.06 | 1a | 6.338 | 3 | CT |
| 28 | Null | 53 | M | White | 102.8 | 28.18 | 1a | 6.121 | 3 | CT |
| 29 | Null | 47 | M | White | 98.5 | 28.78 | 1a | 6.787 | 3 | NA |
| 30 | Null | 48 | M | White | 78.9 | 26.98 | 1a | 6.644 | 3 | TT |
| 31 | SVR | 41 | M | White | 92.7 | 32.08 | 1a | 7.079 | 2 | CC |
| 32 | SVR | 54 | M | White | 88.1 | 28.44 | 1a | 5.215 | 3 | CC |
| 33 | SVR | 45 | M | White | 119.4 | 37.26 | 1a | 5.703 | 3 | TT |
| 34 | SVR | 52 | M | White | 69 | 26.62 | 1a | 6.004 | 2 | CC |
| 35 | SVR | 46 | M | White | 93.5 | 30.88 | 1a | 5.682 | 3 | NA |
| 36 | SVR | 48 | M | White | 100.9 | 30.8 | 1a | 6.669 | 3 | CC |
| 37 | SVR | 54 | M | White | 78.9 | 26.36 | 1a | 5.511 | 3 | CC |
| 38 | SVR | 49 | M | White | 69.9 | 23.9 | 1a | 6.301 | 3 | CC |
| 39 | SVR | 55 | M | White | 80.3 | 26.22 | 1a | 6.898 | 2 | NA |
| 40 | SVR | 46 | M | White | 71.6 | 25.98 | 1a | 6.371 | 2 | CC |
| 41 | SVR | 55 | M | White | 109 | 29.88 | 1a | 6.607 | 3 | CC |
| 42 | SVR | 54 | M | White | 82.6 | 23.12 | 1a | 6.196 | 4 | CT |
| 43 | SVR | 46 | M | White | 92.2 | 31.17 | 1a | 6.481 | 2 | CT |
| 44 | SVR | 51 | M | White | 82.6 | 26.07 | 1a | 5.905 | 3 | CC |
| 45 | SVR | 45 | M | White | 97.2 | 32.1 | 1a | 6.465 | 2 | CT |
| 46 | SVR | 44 | M | White | 83.1 | 25.94 | 1a | 6.233 | 3 | CT |
| 47 | SVR | 42 | M | White | 73.5 | 23.73 | 1a | 5.681 | 3 | TT |
| 48 | SVR | 46 | M | White | 95.3 | 30.77 | 1a | 6.158 | 3 | CT |
| 49 | SVR | 44 | M | White | 112.6 | 34.37 | 1a | 6.493 | 4 | CT |
| 50 | SVR | 45 | M | White | 103.5 | 33.41 | 1a | 5.833 | 3 | CT |
| 51 | SVR | 50 | M | White | 91.9 | 28.05 | 1a | 6.217 | 4 | CC |
| 52 | SVR | 46 | M | White | 100.4 | 33.94 | 1a | 7.146 | 4 | CC |
| 53 | SVR | 55 | M | White | 89.8 | 25.14 | 1a | 6.754 | 4 | CC |
| 54 | SVR | 49 | M | White | 85.9 | 29.04 | 1a | 6.626 | 4 | TT |
| 55 | SVR | 52 | M | White | 81.9 | 25 | 1a | 5.655 | 3 | NA |
| 56 | SVR | 46 | M | White | 87.3 | 26.65 | 1a | 5.820 | 3 | CT |
| 57 | SVR | 41 | M | White | 102.3 | 26.63 | 1a | 6.072 | 4 | CT |
| 58 | SVR | 47 | M | White | 119.6 | 38.18 | 1a | 5.858 | 3 | CC |
| 59 | SVR | 44 | M | White | 88.2 | 30.52 | 1a | 6.908 | 2 | CC |
| 60 | SVR | 48 | M | White | 66 | 25.15 | 1a | 6.769 | 4 | CC |

**Table S1.** An overview of demographic, virological, disease status and genetic information of 60 patients enrolled in the study. Null, null responder; SVR, sustained virological response; M, male, BMI, body mass index; IS, Ishak scores; HCV RNA titers were expressed as log values; IL28B genotype was based on reference single nucleotide polymorphism (SNP) site rs129798; NA, not available.
