# Supplementary material for: High-Resolution Quantification of Hepatitis C Virus Genome-Wide Mutation Load and Its Correlation with the Outcome of Peginterferon-Alpha2a and Ribavirin Combination Therapy
Source: PLoS One. 2014 Jun 20;9(6):e100131. doi: 10.1371/journal.pone.0100131 (PMC4065037; doi:10.1371/journal.pone.0100131)
Supplement: Table S2 — Results of BaTS analysis based on either nearly full-length HCV 1a genome (8817 bp) or genome scan. (DOCX) [file pone.0100131.s005.docx]

**Table S2.** Results of BaTS analysis based on either nearly full-length HCV 1a genome (8817 bp) or genome scan. All 56 patients were divided into two groups by the mean value of the mutation load and the analysis was conducted within each HCV genotype 1a clade. The p values of both the association index (AI) and the parsimony score (PS) less than 0.01 were considered as having statistical significance in terms of mutation load-dependent phylogenetic clustering. Under this setting, only two domains, #34 and #39 respectively located in the HCV NS5a and NS5b genes, showed consistently significance in the clades 1 and 2, indicating their role in the magnitude of HCV population mutation load.
